# Supplementary material for: Cyclo(l-Pro–l-Leu) of Pseudomonas putida MCCC 1A00316 Isolated from Antarctic Soil: Identification and Characterization of Activity against Meloidogyne incognita
Source: Molecules. 2019 Feb 20;24(4):768. doi: 10.3390/molecules24040768 (PMC6412658; doi:10.3390/molecules24040768)
Supplement: Supplementary file 1 [file molecules-24-00768-s001.pdf]

Supplementary information

Cyclo(L-Pro–L-Leu) of *Pseudomonas putida* MCCC 1A00316 Isolated from Antarctic Soil: Identification and Characterization of Activity against *Meloidogyne incognita*

Yile Zhai [1], Zongze Shao [2], Minmin Cai [1], Longyu Zheng [1], Guangyu Li [2], Ziniu Yu [1] and Jibin Zhang [1,*]

[1] State Key Laboratory of Agricultural Microbiology and National Engineering Research Center of Microbe Pesticides, College of Life Science and Technology, Huazhong Agricultural University, Wuhan, 430070, China; zhaiyile1991@163.com (Y.Z.); cmm114@mail.hzau.edu.cn (M.C.); ly.zheng@mail.hzau.edu.cn (L.Z.); yz41@mail.hzau.edu.cn (Z.Y.)

[2] Key Laboratory of Marine Biogenetic Resources, Third Institute of Oceanography, State Oceanic Administration, Xiamen 361005, China; shaozz@163.com (Z.S.); mccc_ligy@163.com (G.L.)

* Correspondence: zhangjb05@163.com; Tel/Fax: +86-27-87287254

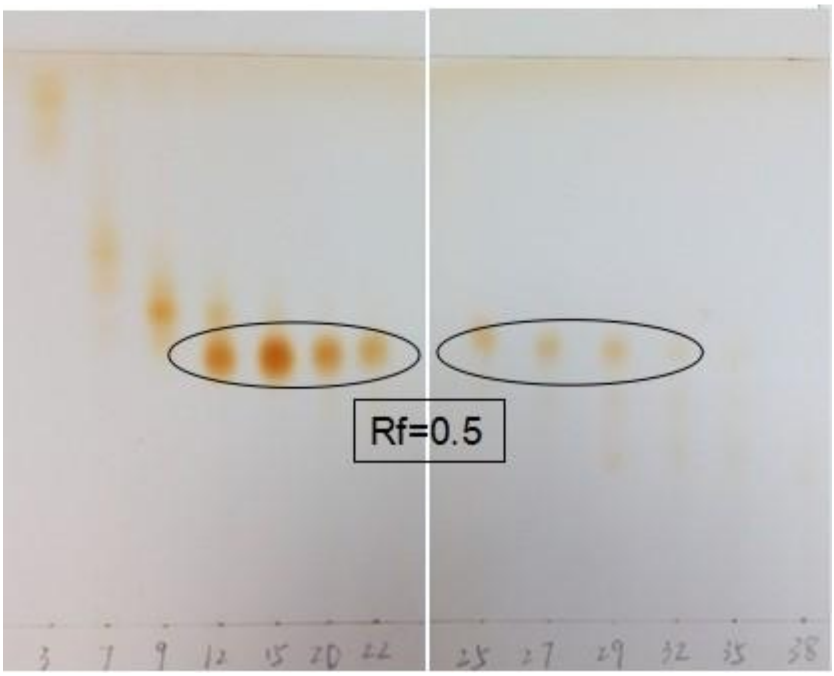

Figure S1 TLC plates of 38 fractions (Fr 1-38) obtained from a large silica gel column. The desired fraction is the one with a Retention Factor Value (Rf) of 0.5.

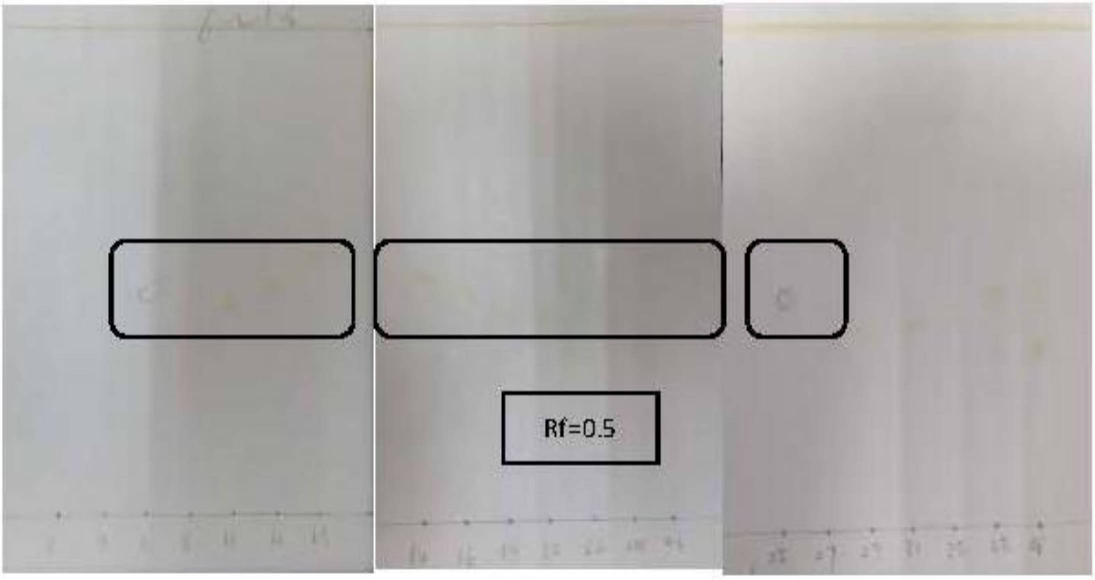

Figure S2 TLC plates of 35 fractions (Fr 1-35) obtained from F1-1 using a small silica gel column. The desired fraction is the one with a Retention Factor Value (Rf) of 0.5.

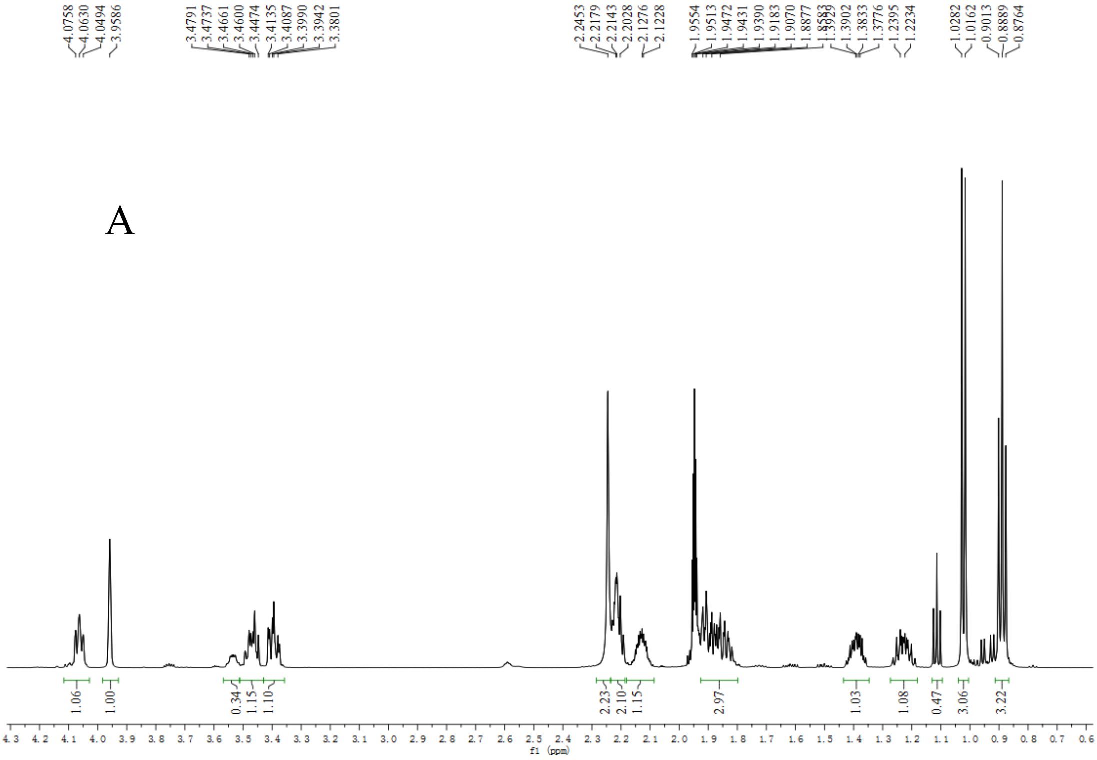

Figure S3. *Cont.*

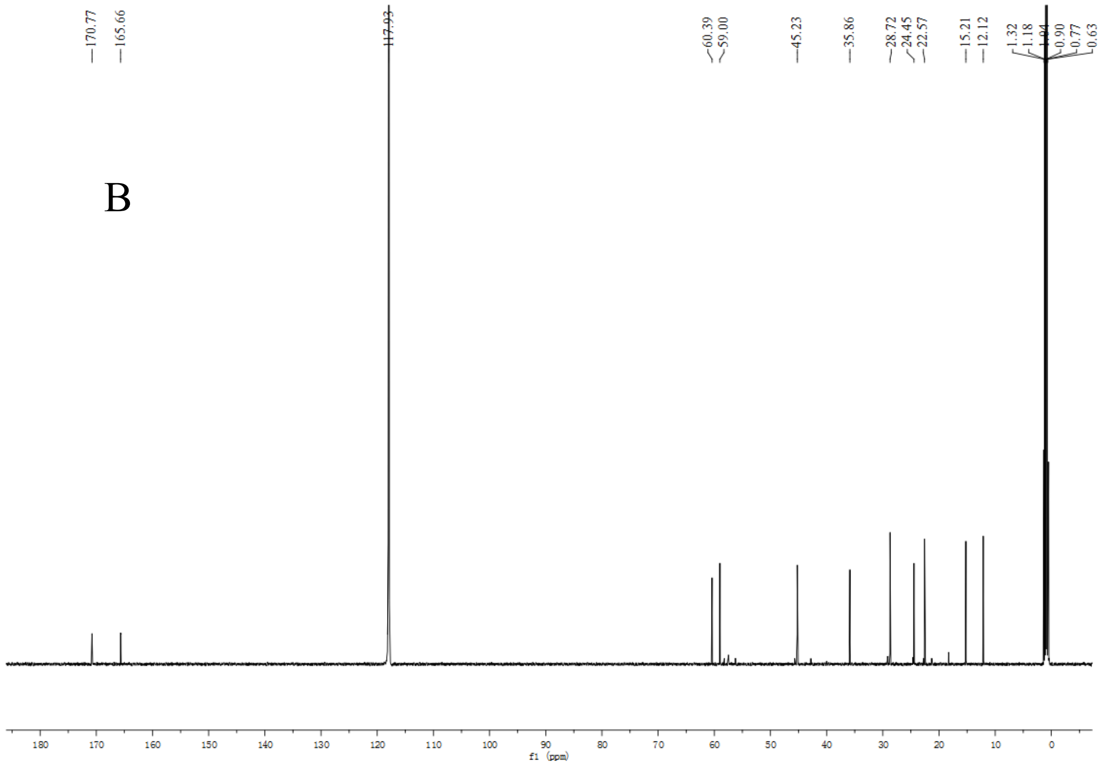

Figure S3 ^{1}H-NMR spectrum of compound P3 in CD_3CN at 600 MHz (A), ^{13}C-NMR spectrum of compound P3 in CD_3CN at 150 MHz (B).

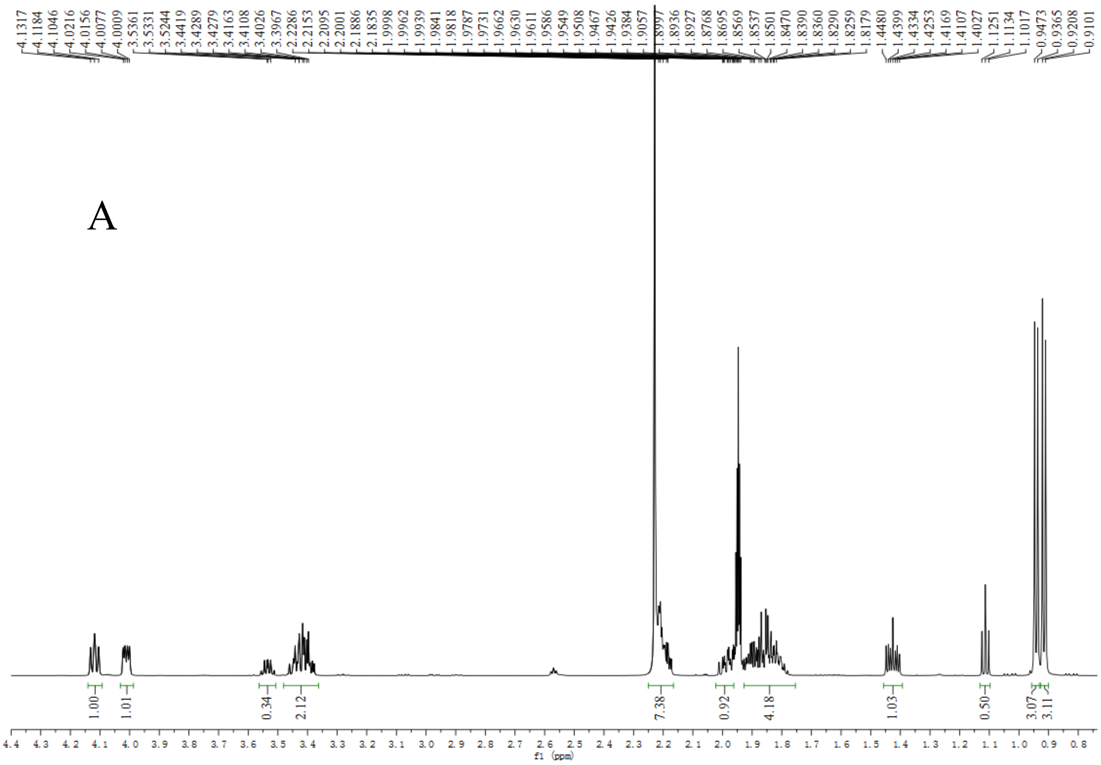

Figure S4. *Cont.*

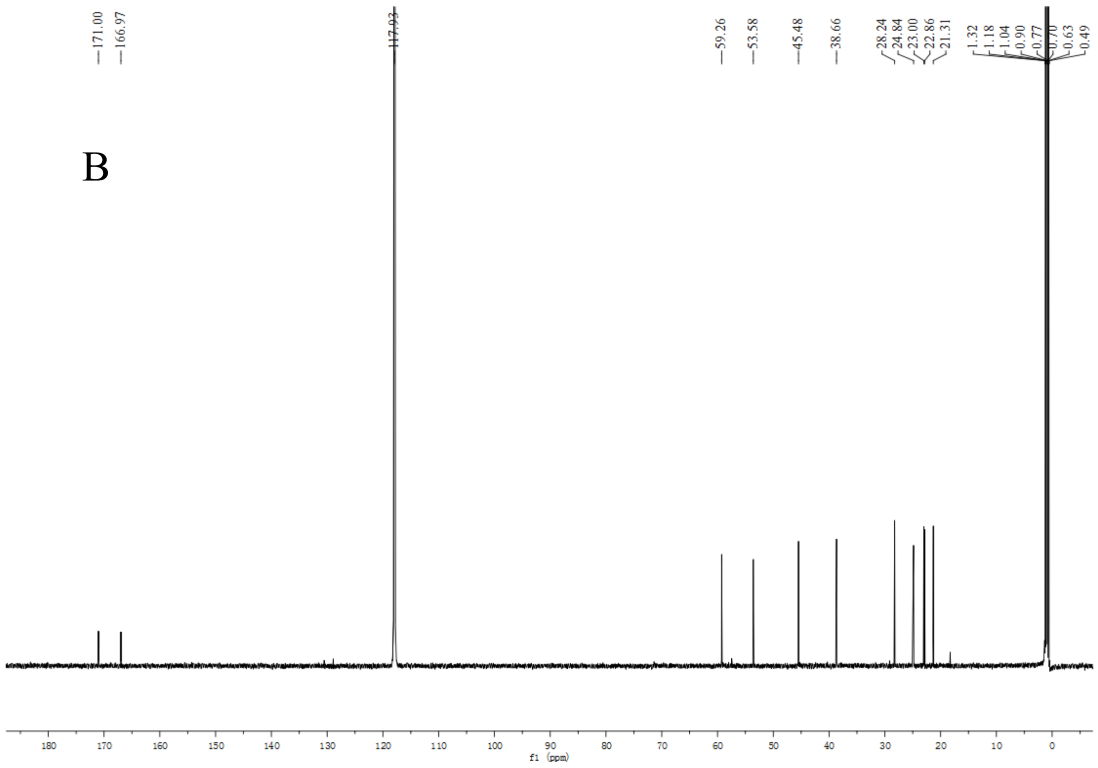

Figure S4 ^{1}H-NMR spectrum of compound P4 in CD_3CN at 600 MHz (A), ^{13}C-NMR spectrum of compound P4 in CD_3CN at 150 MHz (B).

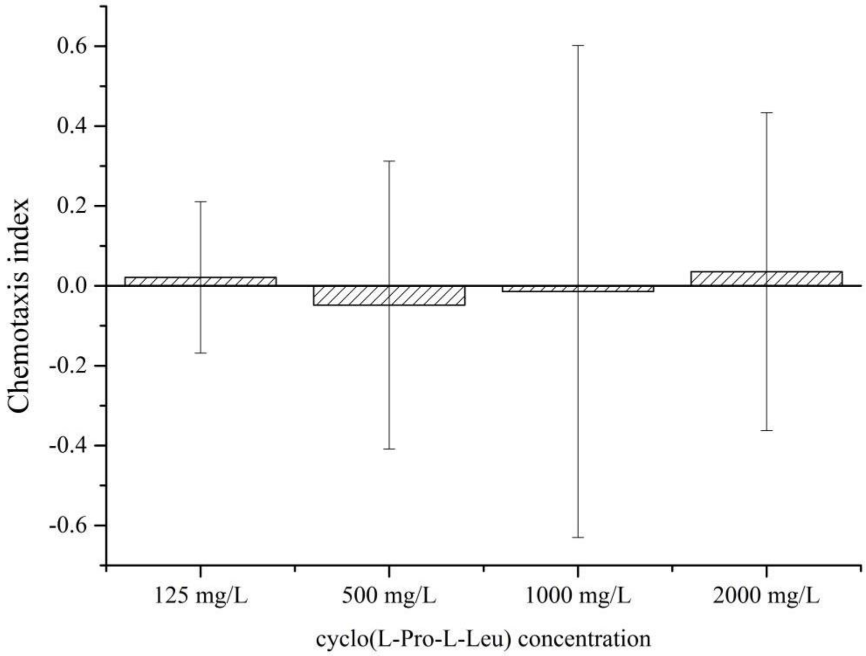

Figure S5. Chemotaxis of *M. incognita* J2 to cyclo(L-Pro-L-Leu). Values with the * do not differ from each other at $P < 0.05$, they means the side of the number of attracting or avoiding nematodes is significantly different from another side; bars indicate the standard error of the means (n = 3).
